# MLTrends: Graphing MEDLINE term usage over time

**Published:** 2010-01-25

**Authors:** Gareth A Palidwor, Miguel A Andrade-Navarro

**Affiliations:** 1 Ottawa Hospital Research Institute, 501 Smyth Road, Ottawa, Ontario, Canada, K1H 8L6; 2 Computational Biology and Data Mining group, Max Delbrück Center for Molecular Medicine Robert-Rössle-Str. 10, 13125 Berlin, Germany

## Abstract

The MEDLINE database of medical literature is routinely used by researchers and doctors to find articles pertaining to their area of interest. Insight into historical changes in research areas and use of scientific language may be gained by chronological analysis of the 18 million records currently in the database, however such analysis is generally complex and time consuming. The authors’ MLTrends web application graphs term usage in MEDLINE over time, allowing the determination of emergence dates for biomedical terms and historical variations in term usage intensity. Terms considered are individual words or quoted phrases which may be combined using Boolean operators. MLTrends can plot the number of records in MEDLINE per year whose titles or abstracts match each queried term for multiple terms simultaneously. The MEDLINE database is stored and indexed on the MLTrends server allowing queries to be completed and graphs generated in less than one second. Queries may be performed on all titles and/or abstracts in MEDLINE and can include stop words. The resulting graphs may be normalized by total publications or words per year to facilitate term usage comparison between years.This makes MLTrends a powerful tool for rapid evaluation of the evolution of biomedical research and language in a graphical way. MLTrends may be used at: http://www.ogic.ca/mltrends

## Introduction 

MEDLINE is a medical and life sciences bibliographic database containing more than 18 million records ranging back to 1950. It is available over the web via the PubMed web interface and is an important resource used by researchers and doctors worldwide (1). Besides its primary use as a tool for reference search, MEDLINE mirrors the progress of biomedical research and analysis of the terms used in large numbers of articles can provide insights into the evolution of ideas and terminology in the field (2, 3), which can be of interest not only to researchers but also to educators, journalists, and policy makers. Due to the size and complexity of the MEDLINE database, such analysis can be difficult and time consuming, requiring technical skills not available to the general public.

**Figure 1:**
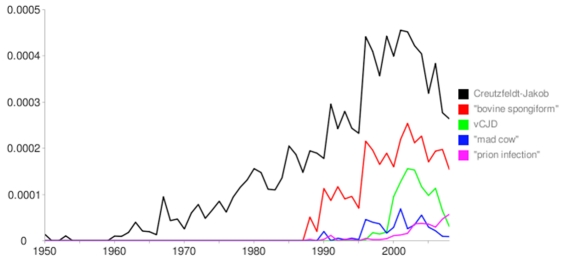
MLTrends output showing the fraction of records in MEDLINE with a given term in their title or abstract versus year of publication.

To simplify the visualization of historical variations in term usage intensity for multiple terms simultaneously we have implemented MLTrends (http://www.ogic.ca/mltrends) (Figure 1). This tool allows users to graph term usage per year in MEDLINE entries. Terms are individual words or quoted phrases which may be combined using Boolean operators. The numbers of publications whose specified fields exactly match each queried term are recorded. MLTrends can plot the number of records in MEDLINE per year matching the query. However, since the number of publications is larger every year, we offer the possibility of normalizing the data by dividing the value in each data point by the number of MEDLINE records in the year. A further normalization option offered is by total number of word instances in the records of the year. This is useful because on the one hand abstracts tend to be longer, and on the other hand most records before the 80s contain just titles. Therefore, normalizing by the number of word instances in all records of the year somehow avoids an artifactual jump.

## Methods 

Apache Lucene, a Java based indexing and search library, is used to index the MEDLINE database for rapid searching. For each record in MEDLINE, all words in the abstract and title are indexed as well as the publication date. Total words in abstracts and titles are counted. The indexing process for the full database takes approximately 12 hours. The resultant index is large (more than 4 gigabytes in size), however searches using the index take just milliseconds. A Java application is used to search the index returning numbers of records that match the search term for every year from 1950 to the present. 

The web interface to the index is written in the Perl programming language, passing queries to the Java search tool described above and generating line graphs using the Google Chart API. Searches may be performed on the title or title and abstract fields of the MEDLINE records, and normalized by either publication count or total word count in the chosen fields. The search interface supports a subset of the standard Lucene query syntax: Boolean search operators OR, AND and NOT are supported between terms and adjacent words or phrases without explicit operators are implicitly OR-ed together. Searches are case insensitive, though the Boolean operators must be upper case to distinguish them from search terms.

Y-axis scale is hits, normalized by documents or words per year. To facilitate comparison of terms with a both high and low usage, graphs can optionally be log-scaled. 

To speed searches and reduce load on the hosting server all query results are cached in a local file, with later duplicate queries using the stored results rather than Lucene.

## Results and Discussion

The utility of visualizing temporal term usage analysis in MEDLINE with MLTrends is illustrated here by exploring the timeline of events surrounding the discovery that a variant of the Creutzfeldt-Jakob disease (vCJD), a human neurodegenerative disease, is caused by consumption of brain tissue from cows affected by Bovine Spongiform Encephalopathy (BSE), and the eventual explanation of transmission by prion infection.

The graph (Figure 1) shows usage over time of the terms Creutzfeldt-Jakob, “bovine spongiform”, vCJD, “mad cow”, and “prion infection”, and largely summarizes the recent history of this research topic highlighting key dates and trends that can guide a literature review. The graph shows that Creutzfeldt-Jakob disease (CJD) has been researched since the 1950s and in fact was known since 1920, before the date of the oldest records currently stored in MEDLINE. In 1988, coincidental with the first use of the term “bovine spongiform” (from BSE) recorded in MEDLINE, an epidemic of the disease happens in the UK and other countries, possibly caused by feeding cattle with processed cow carcasses, and thousands of cows are sacrificed and destroyed. Immediately, a hypothetical connection to human cases of CJD is proposed (4) but remains unproven. This is quickly picked up in the news and the term “mad cow disease” is popularized as a synonym for BSE but has yet to enter the scientific literature (a peak of publications in 1990 mention the term but they are mostly news commentaries in scientific journals (5)).

Following the epidemics, the 1990-1996 period shows an increase in the research on both BSE and CJD. As a result of this effort, multiple evidence that people can be infected from BSE-infected cows is published after October 1996 (6), and the new term vCJD is coined to define a particular form of CJD transmitted from cows affected by BSE, eventually identified as the most common form of CJD. As a result, there is a further increase in publications dealing with these topics during the 1996-2004 period. This may have attracted attention to the study of prion infectivity, and the term “prion infection” exhibits continuous and pronounced growth after the initial description of vCJD. Finally, the decrease in recent years (2005-2008) of the number of manuscripts mentioning vCJD and BSE suggests that either once the mechanisms underlying the disease were known, researchers moved on to other novel unsolved biomedical problems or that the importance of the disease decreased after measures were taken and the number of patients decreased. This type of analysis can clearly show periodic shifts of research efforts from topic to topic.

The effectiveness of MLTrends is limited by term ambiguity in MEDLINE (7). In the example discussed above we could not use the abbreviation BSE as it has multiple meanings in MEDLINE such as breast self-examination, brief spindle episodes, and backscattered electron. 

In most cases, one can get around this problem by considering either alternative related terms (e.g. “bovine spongiform” above) or using Boolean operators (e.g. bse AND bovine). The tool is quick enough to allow experimenting with different terms in a short time. In any case, this is an exploratory tool to suggest trends and we recommend testing the results by examining selected examples. For example, querying PubMed with “BSE AND 1980:1985 [Entrez Date]” quickly shows that the abstracts in that period do not deal with mad cow disease.

This problem is partially solved in other methods at the expense of power and speed. For example, Anne O’Tate (8) can refine the user’s query by finding associated MeSH terms that are attached to MEDLINE entries by PubMed annotators, e.g. for BSE it will offer as option to focus the search on abstracts with the MeSH term for bovine spongiform encephalopathy. Anne O’Tate however, cannot produce graphs for multiple terms at once, it is limited to 25000 abstracts, and may take more than two minutes to respond to a query for those. This simply reflects that Anne O’Tate and MLTrends have a different focus. 

There are other methods available as online tools that offer analysis with capabilities of displaying term usage per year, but, unlike Anne O’Tate, current versions are not supported by bibliography. Of those, GoPubMed (9) offers a similar extension of the query by using MeSH terms, and a histogram of one term at a time, but with a limit on 100,000 papers. Other systems exist that need to query PubMed and therefore take a long time to respond: MEDLINE Trend (10) will only plot one term at a time and will take 30 seconds for queries with many hits; PubReMiner (11) is limited to 6000 abstracts and will take more than 10 minutes to produce an output; TiPS beta (12) has the best graphics and allows searching up to eight terms, but needs minutes for queries with common terms.

In summary, a fundamental difference between MLTrends and all the systems mentioned above, including NCBI’s Entrez, is that MLTrends indexes all words, including stop words normally excluded from indexing such as “it” and “and”. Therefore, searches with strings of words such as “we found” and “it was found,” that return no results in all the systems mentioned above, can be obtained in seconds. For example, this allows observing the increasing trend of using the active form versus the passive form in scientific language. MEDLINE is also a repository of linguistic information (13) and MLTrends can put such data in a temporal context.

## Conclusion

As shown above, MLTrends can be used to quickly and easily describe the evolution of biomedical research and language in a graphical way. It is intended to be usable by non-researchers and the general public to allow rapid evaluation of term usage evolution. While generation of these graphs is now easy and quick, interpretation should be done with care, since terms with multiple meanings can confound results.

## Acknowledgements

MAA acknowledges funding support from the Helmholtz Alliance on Systems Biology. The authors are grateful to Andre Vellino of the Canadian National Research Council, Neil R. Smalheiser and an anonymous reviewer for providing useful criticisms and suggestions.
